# Maleic Anhydride Polylactic Acid Coupling Agent Prepared from Solvent Reaction: Synthesis, Characterization and Composite Performance

**DOI:** 10.3390/ma15031161

**Published:** 2022-02-02

**Authors:** Helena Oliver-Ortega, Rafel Reixach, Francesc Xavier Espinach, José Alberto Méndez

**Affiliations:** Group LEPAMAP-PRODIS, Department of Chemical Engineering, University of Girona, 17003 Girona, Spain; rafel.reixach@udg.edu (R.R.); francisco.espinach@udg.edu (F.X.E.); jalberto.mendez@udg.edu (J.A.M.)

**Keywords:** polylactic acid (PLA), coupling agent, biocomposites

## Abstract

In the present work, a functionalization of polylactic acid (PLA) has been carried out to anchor maleic anhydride onto the main polymer chain to promote improvement in the compatibility of this polymer matrix with cellulose fibres. Low-molecular-weight PLA has been reacted with maleic anhydride following different procedures: a bulk reaction in an internal mixer and a solution reaction. The presence of oxygen during bulk processing did not allow for functionalization, guiding the reaction towards a decrease in the molecular weight of the material. On the contrary, a controlled reaction under an inert atmosphere in the presence of dioxane as the solvent, at reflux temperature, led to the functionalization of the polymer reaching different yields depending on the percentage of radical initiator and maleic anhydride added and reaction time. The yield of functionalization has been monitored by acid number titration as well as 1H NMR, with optimal yield values of functionalization being up to 3.5%. The PLA-functionalized formula has been used to make commercial PLA compatible with cellulose fibres derived from a thermomechanical treatment. The addition of 10% *w*/*w* of fibres to PLA increases the ultimate tensile strength (UTS) of PLA by up to 15%. The incorporation of 4 *w*/*w* of the already-functionalized coupling agent to the composite produces improvements in UTS of up to 24% regarding PLA, which confirms the functionalization from a performance point of view.

## 1. Introduction

Cellulose-reinforced polymer composite materials have been promoted as an alternative to common plastic or synthetic fibre-reinforced composites [1,2]. Cellulose is a bio-based polymer, with high abundance in nature and moderate mechanical properties in comparison with other reinforcement fibres. Nonetheless, its lower density and lower mechanical performance allow or require higher quantities of the composite material. Thus, it leads to competitive composites with lower polymer fractions and similar weights to other composites [3]. In addition, cellulose composites are mechanically recyclable, while this is almost impossible for synthetic fibres, such as glass fibres, due to their fragility [4].

Nevertheless, the high polarity of these fibres represents a disadvantage when polymers are reinforced, because polymers are generally highly nonpolar materials. Different strategies have been used to avoid this phenomenon. One example is the use of polar polymers such as polyvinyl alcohol, thermoplastic starch, or polyamides [5,6,7]. These polymers have a higher polarity due to the presence of functional groups with the capacity to establish H-bonds, a strong intermolecular interaction. Another option is the use of coupling agents, which are modified polymers containing groups that, in presence of the hydroxyl groups of cellulose, react, establishing covalent bonds with cellulose. At the same time, the coupling agent bonded to the cellulose fibres diffuses in the polymer matrix, enhancing the dispersion of the fibres [8]. This is the case in the successful example of maleic-anhydride-grafted polypropylene (MAPP) or polyethylene (MAPE) used in polypropylene and polyethylene composites (PP and PE). Nonetheless, these coupling agents need to be prepared from the same polymer matrix as used in the composite material to ensure the best performance. The use of different polymers could reduce or completely inhibit the objective of the coupling agent due to polymers not blending properly [9,10,11]. Thus, the synthesis of a coupling agent for each specific polymer is necessary, such as in the case of polylactic acid (PLA). 

**Figure 1:**
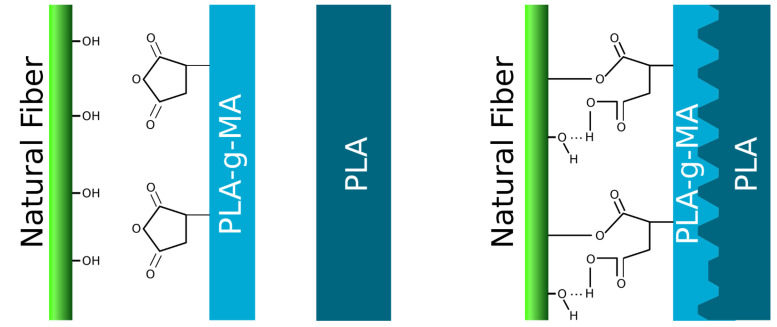
Grafting PLA mechanism in PLA-reinforced natural fibre composites. Figure produced with Inkscape software.

PLA is, nowadays, the most promising biobased, biodegradable and compostable polymer to substitute those based on petrol [12,13,14,15]. These properties, along with the mechanical performance of the polymer, allow it to be a sustainable replacement for a large variety of uses and applications, from packaging to structural applications. Moreover, although it is biodegradable, the conditions for its biodegradation or compostability require moderate and controlled temperature and humidity due to the high stability of the polymer in ambient conditions [16]. Nevertheless, composite materials made from PLA could be used in high mechanical property applications and could reduce its price by reducing the polymer content with a cheap component. However, the miscibility of PLA and cellulosic fibres is limited [17]. PLA is a polyester, and the interaction of the ester bond is poor. Thus, the polarity of this polymer is slightly higher than completely nonpolar polymers such as PP or PE, but still far from polyamides. This slight miscibility has led to improvements in the mechanical properties that have been observed [18,19,20], however, the reinforcement effect is lower than expected in comparison with composite materials from nonpolar matrixes and those prepared using a coupling agent in the formulation [21]. In this sense, effort has been made to prepare a coupling agent for PLA. Usually, the preparation of the coupling agent has been conducted by reactive extrusion such as MAPP and MAPE, producing a grafted PLA (PLA-g-MA). This is expected to work via the mechanism shown in Figure 1. However, the thermal and oxidative susceptibility of PLA is higher than PP and PE and it generally leads to poor grafting degrees (GD) and a drastic loss of molecular weight. Other approaches, such as melt functionalization [22], have been explored, and, the grafting degrees were lower than the ones obtained by reactive extrusion. 

In this work, the authors propose the preparation of a coupling agent for PLA by its grafting in a solvent with inert atmosphere. The solvent approach requires lower temperatures in comparison with the previous ones, as it is not necessary to melt the polymer. This avoids thermal degradation in the polymer. In addition, it is easily purified by precipitation directly from the solvent reaction mixture. For comparison, a coupling agent was also produced from reactive extrusion. Once the solvent system showed efficiency in the grafting, parameters were modified to obtain the higher grafting degree and then tested in a 10% reinforced composite material. 

## 2. Materials and Methods

### 2.1. Materials

PLA 3001D (Nature Works, Naarden, The Netherlands), a PLA brand with low molecular weight (84,500 Da of Mn), was used as a polymer for the preparation of the coupling agent. Maleic anhydride (MA), dicumyl peroxide (DCP), and calcium hydride (CaH_2_) were used for the synthesis of the coupling agent. Deuterated chloroform (CDCl_3_) used for the nuclear magnetic resonance (NMR) and tetrahydrofuran (THF) for the gel permeation chromatography (GPC) characterization were purchased from Sigma Aldrich (Madrid, Spain). Dioxane, dichloromethane, methanol, potassium hydroxide, and hydrochloric acid (37%) were supplied by Scharlau (Sentmenat, Spain).

PLA Ingeos Biopolymer 3251D (Nature Works, Naarden, The Netherlands), described as PLAc, was used as a polymer matrix for the fibre-reinforced composites. The reinforcement fibres were thermomechanical pulp from Norske Skog Saugbrugs.

### 2.2. Methods

#### 2.2.1. Synthesis of the Coupling Agent

The synthesis of the coupling agent was carried out following two different methodologies: reactive extrusion and its reaction in a solvent system. The direct reaction in the extrusion equipment was performed in an internal mixer (Brabender^®^ plastograph mixing machine, Duisburg, Germany) at 40 rpm and 190 °C in the mixing chamber. Initially, PLA, previously dried in a vacuum line at room temperature over 48 h to avoid hydrolytic degradation, was melted in the mixing chamber. Afterward, a 0.25% *w*/*w* PLA of DCP was added to the mixing chamber and mixed with the polymer for 3 min. Finally, the MA was added and reacted for 5 min. Three different quantities of MA were tested: 3 (M1), 4.5 (M2), and 6% *w*/*w* PLA (M3).

**Figure 2:**
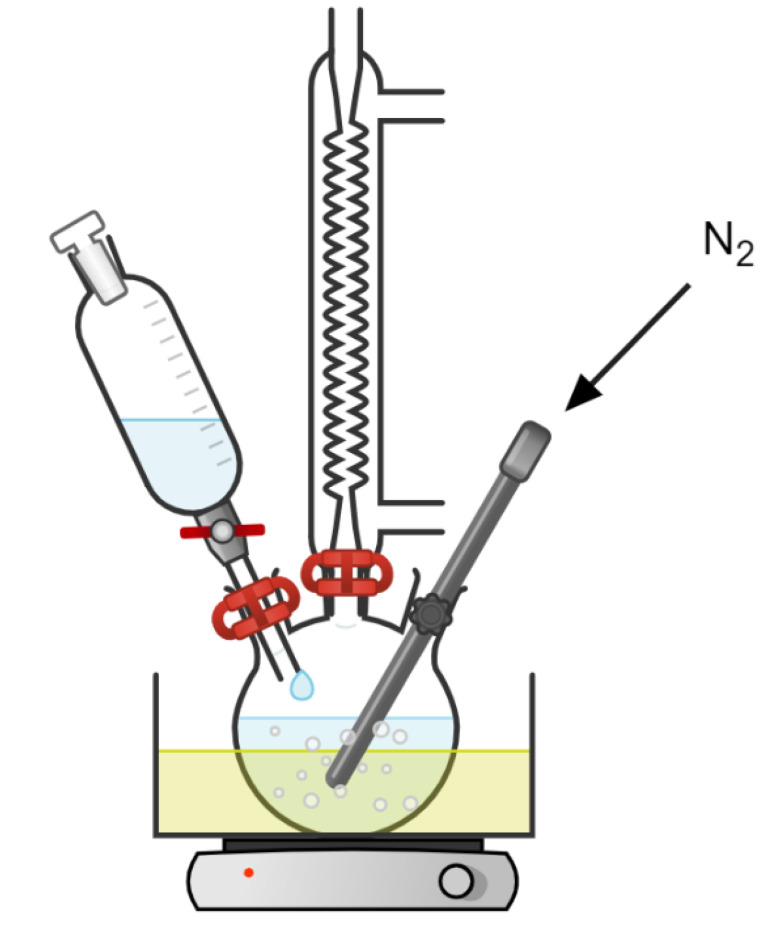
Scheme of the solvent system reaction. Figure produced with Chemix application (Codelite Ltd., London, UK).

For the grafting reaction in the solvent system, the dried PLA was solved in previously dried dioxane and purged with N_2_ in a round-bottom flask. To dry the dioxane, two spoons of calcium hydride were added in 250 mL of dioxane and stirred for 24 h at room temperature to eliminate solved water. Afterward, dioxane was distilled and directly used for the synthesis. Once PLA was solved, MA was added to the flask until it was solved. The process is performed in N_2_ atmosphere. DCP was solved in 80 mL of dried dioxane and placed in a dropping funnel and was also purged with N_2_. Then, DCP was slowly added to the round bottom-flask containing PLA and MA. During the addition, the system is maintained in an N_2_ atmosphere. After the drop addition was finished, the N_2_ purge was maintained for 4 h (Figure 2).

**Figure 3:**
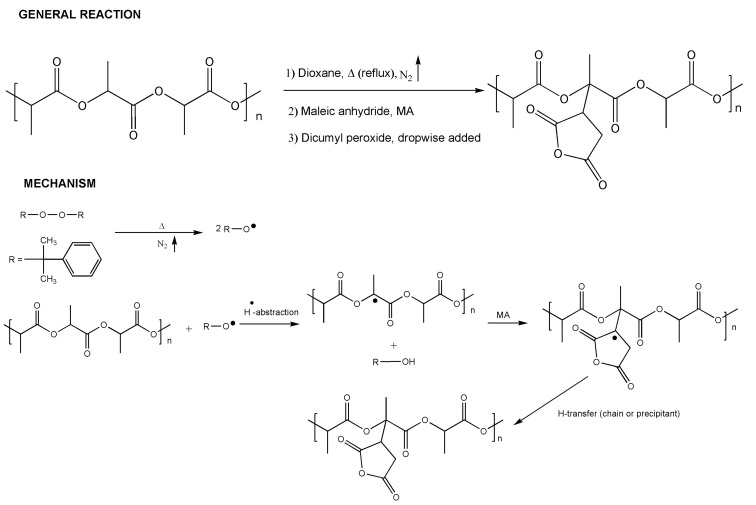
Scheme of the solvent system reaction.

The proposed mechanism for the reaction between PLAs and MA using DCP as the initiator is described below (Figure 3). 

Different reaction times, MA and DCP ratios were studied for the synthesis of the coupling agent. The first sample in the solvent system was performed with a 2% *w*/*w* of DCP regarding PLA content, 20% *w*/*w* of MA also regarding PLA content, and 4 h (M4). This sample was performed without N_2_ atmosphere. Nonetheless, it is well-known that O_2_ could inhibit the reaction. Thus, a test with the same quantities of reagents but a purge of N_2_ to keep the inert atmosphere was performed (M5). The effect of the DCP percentage was tested with the same reaction but using a 1% *w*/*w* PLA of DCP. The effect of the MA content was tested by performing the reaction with 5, 10, and 20% *w*/*w* PLA of MA, and 1% *w*/*w* PLA of DCP for 4 h. Finally, the time of reaction was adjusted, performing the reaction in the better conditions of DCP and MA at 4, 8, 24, and 96 h. Table 1 summaries all of the synthesis conditions:

After the reaction was performed, the grafted PLA was precipitated and purified in cold methanol. PLA was collected by filtration and dried in a vacuum line at room temperature. 

#### 2.2.2. Characterisation of the Coupling Agent

The synthesized coupling agents were characterized using acid number analysis (AN), gel permeation chromatography (GPC), nuclear magnetic resonance (^1^H-NMR), and differential scanning calorimetry (DSC). The acid number test led to the calculation of the number of acid groups in the PLA chains by indirect titration. A quantity of 1.0 g of MA-modified PLA was solved in dichloromethane using a ground-necked Erlenmeyer flask connected to a condenser to avoid dichloromethane evaporation. Once PLA was dissolved, 25 mL of KOH 0.01 N was added to react with the acid groups. The excess KOH was measured by reacting with HCl 0.01 N. Thus, AN was determined as:

(1) $$\mathrm{AN}=\frac{\left(V_{\mathrm{KOH}}\cdot C_{\mathrm{KOH}}-V_{\mathrm{HCl}}\cdot C_{\mathrm{HCl}}\right)\cdot M_{\;\mathrm{KOH}}}{W_{p}}$$

where M is the molecular weight of the potassium hydroxide and Wp is the polymer weight. The AN test has been performed in triplicate. The AN led to calculating the grafting degree (GD) in the polymer in comparison with the unmodified PLAs, and considering the Mw of KOH, the M of the maleic anhydride:

(2) $$\mathrm{GD}\;\left(\%\right)=\frac{\left({\mathrm{AN}}_{\;s}-{\mathrm{AN}}_{\;\mathrm{PLA}}\right){*M}_{\mathrm{MA}}}{M_{\;\mathrm{KOH}}\cdot 2\cdot W_{p}}\cdot 100$$

The molecular weight of the samples was analysed using gel permeation chromatography (GPC). The chromatography equipment was equipped with a Styragel HR 5 E column, from Waters (Waters Chromatography, Cerdanyola del Vallés, Barcelona, Spain), and a refractive index detector. The eluent was THF and polystyrene standards were used for GPC calibration. The temperature of the test and column was room temperature. A minimum of 2 repetitions was performed to confirm the molecular weight. The chemical structure of PLA-grafted MA was checked by ^1^H nuclear magnetic resonance in a Bruker Ultrashield ASCEND Nanobay (Bruker, Madrid, Spain) with a frequency of 400 MHz, atb room temperature, and using CDCl_3_ as solvent. Finally, differential scanning calorimetry analysis (DSC) was performed to analyse the thermal behaviour of the coupling agent. A Mettler Toledo DSC822e calorimeter (Mettler Toledo, l’Hospitalet de Llobregat, Spain) was used following ASTM E 1269.01 standard specification. Around 10 mg of sample was introduced in an aluminium pan. The samples were initially heated from 30 to 200 °C to erase their thermal history. Afterward, the samples were cooled and heated again using the same temperature range. All runs were performed at heating or cooling rates of 10 °C/min under 40 mL/min flow of nitrogen atmosphere.

#### 2.2.3. Composite Materials’ Preparation

The mechanical efficiency of the coupling agent was analysed by the production of a 10% cellulosic reinforced composite material. The composite was prepared in a Gelimat Kinetic Mixer (Ramsey, NJ, USA). The polymer (PLAc) and the coupling agent were added at a low speed (300 rpm) in the mixing chamber. At the same speed, the fibre was later added to the chamber, and then the speed was increased to 2500 rpm. Once the temperature of the chamber achieved 200 °C, the material was discharged, cooled down, and milled to obtain the adequate size for the transformation process. 

#### 2.2.4. Composite Materials’ Tensile Characterization

Samples for mechanical testing were obtained using injection moulding using an Aurburg 220 M 350–90U equipment (Aurburg, Loßburg, Germany). The temperature profile was 180–190–200–210 °C and the pressures ranged from 300 to 350 bars. Samples were mechanically characterized for tensile properties in a DTC-10 Universal testing machine (IDMtest, New York, NY, USA) equipped with a 5 kN load cell and a deformation speed of 2 mm/min. The Young modulus was calculated using an extensometer (Hounsfield H2.5 K-S, Tinius Olsen Ltd., Salfords, Surrey, UK) following the standard specifications. Different specimens were used for the tensile strength and Young’s modulus. The deformation was obtained from the tensile strength test and the values correspond to the deformation at the breaking point. Previously to the tests, samples were conditioned at 23 °C and 50% relative humidity for 48 h in a Dycometal conditioning chamber following ASTM D618 standard. A minimum of five specimens of each sample has been tested for each analysis. 

## 3. Results and Discussion

### 3.1. Synthesis of the Coupling Agent

#### 3.1.1. Reactive Extrusion 

The AN and the GD of the samples were analysed by indirect titration. The results are shown in Table 2.

The results showed a slight increment of the neat polymer AN when the reactive extrusion was performed. This increment could be understood as grafting of 0.16, 0.17, and 0.18%, respectively for all the samples. In PP and grafted polymers, the grafting degree and the acidic number are not high [23,24]. Nonetheless, the slight increment in the AN in PLA could be related to the degradation of PLA, increasing the acid end groups available due to the reduction in the polymer chains. 

**Figure 4:**
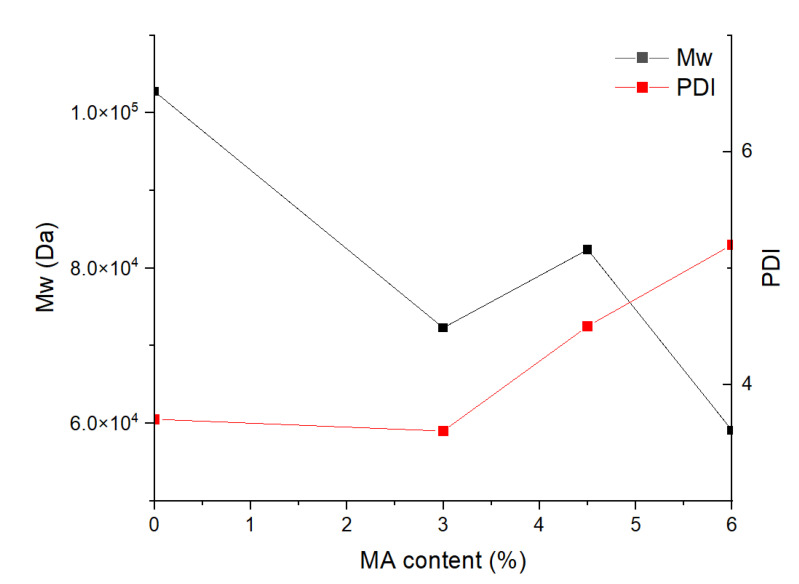
GPC results from the PLA and reactive extrusion samples: weight-average molecular weight (Mw) in black and polydispersity index (PDI) in red.

The GPC led us to determine if there was any change in the molecular weight of these polymers and the chains distributions. PLA is a condensation polymer that tends to have a not-so-high average molecular weight (Mn) and weight-average molecular weight (Mw) and broad distributions and polydispersity index (PDI) over 2. In the synthesized sample, the PLA used was a low-molecular-weight PLA. It is expected that if degradation has been found during the reactive extrusion, the samples will show a clear reduction in the Mn and Mw and an increase in the PDI. Other authors have confirmed this by DSC or tensile strength tests [25], however Mw analysis is more accurate. Results are shown in Figure 4.

The reactive extrusion produced a reduction in the Mw even at low MA contents. The reduction is higher when the MA content is raised in the grafting, and achieves the lowest value of Mw for the grafted PLA synthesized using 6% *w*/*w* MA. The value of Mw is almost half compared to that of neat PLA. Moreover, the PDI results increased drastically up to 3% of MA used, indicating a broader distribution of the PLA chain which could be related to the huge degradation of the samples. These results led us to consider that all the increment observed in the AN is related to the degradation of the polymer chains instead of the grafting of PLA. Moreover, the degradation observed in the PLA chains will affect the mechanical properties of the PLA and could produce a negative effect in the composite materials if the quantity of coupling agent necessary for the compounding became higher. 

**Figure 5:**
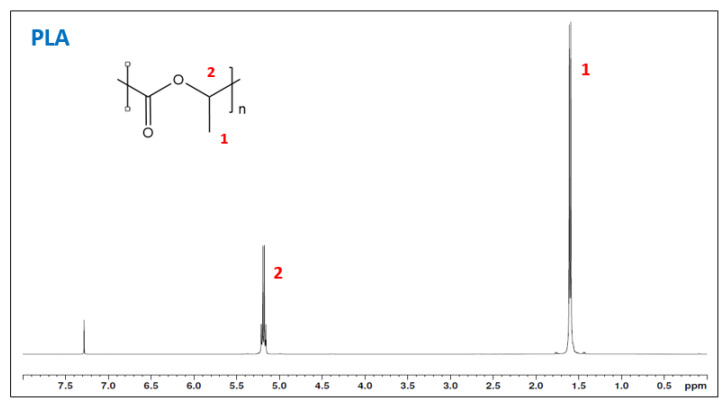
H^1^-NMR of PLA.

**Figure 6:**
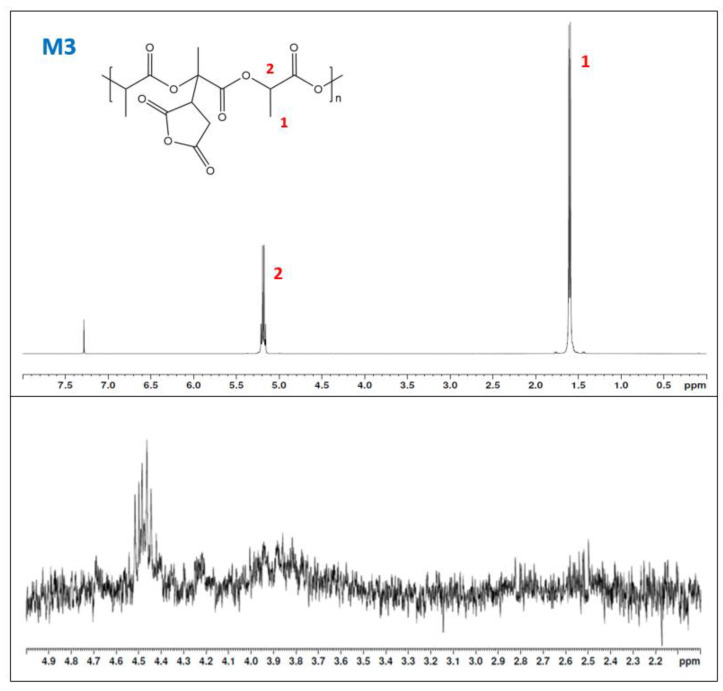
H^1^-NMR of sample M3. The lower part of the figure for sample M3 is a magnification of the range from 2 to 5 ppm to observe the apparition of the hydrogens from the succinic ring.

Nonetheless, the AN could be quite imprecise to determine the grafting, as polymer degradations affect it. Thus, ^1^H-NMR is more accurate to evaluate the chemical structure and determine if there was some grafting in the samples. In addition, although degradation is obtained, it is interesting to characterize the chemical structure of the grafted PLA and determine if the grafting reaction has been produced. The reactive extrusion sample M3 and PLA were characterized using ^1^H-NMR (Figure 5 and Figure 6). The neat PLA (Figure 5) presents two main peaks at 1.6 ppm corresponding to the three hydrogens from the methyl group and another one corresponding to the hydrogen bonded to the α-carbon. The grafted samples showed the same peaks. Figure 4 shows the spectra of sample M3. Magnification from the 2 to 5 ppm region is performed in sample M3 (lower part of the figure) to observe the apparition of the methine and methylene (around 2.3 ppm and 3.7 ppm, respectively), but nothing was realized [26]. Thus, no grafting was observed from the reactive extrusion and the methodology was discarded and moved to the solvent system. The lack of grafting has been previously described as a poor chemical activity due to the PLA chemical structure [27]. Nonetheless, it could be related to the oxygen present in the mixing chamber which could react with the radicals formed and reduce the effectivity of the reaction while enhancing the degradation effect also caused by the extrusion equipment. 

#### 3.1.2. Solvent Synthesis Methodology

Initially, synthesis was performed in air and nitrogen atmospheres in independent experiments, to evaluate the effect of oxygen in the reaction. It is well-known that oxygen acts as a radical catcher and could inhibit the reaction. Thus, samples M4 and M5 were performed with the same composition, 2% DCP and 20% of MA for 4 h, but in M5 N_2_ purge to ensure oxygen elimination was kept constant during the reaction. The AN results are shown in Table 3. 

The results of the AN showed a slightly higher result for M4 and a clear increase in the result of the M5. These increments reported higher GD than those observed previously with the extrusion reaction, and they increased significantly when an inert atmosphere was maintained in the reaction. The absence of oxygen could explain the improvement in the GD of sample M5, as the oxygen does not interact with the radicals. Nonetheless, the increment in the AN could be also related to the degradation of the PLA chains as above observed, although the synthesis conditions are gentle in comparison with the reactive extrusion. The Mw of both samples was analysed by GPC and the results are shown in Table 4.

**Figure 7:**
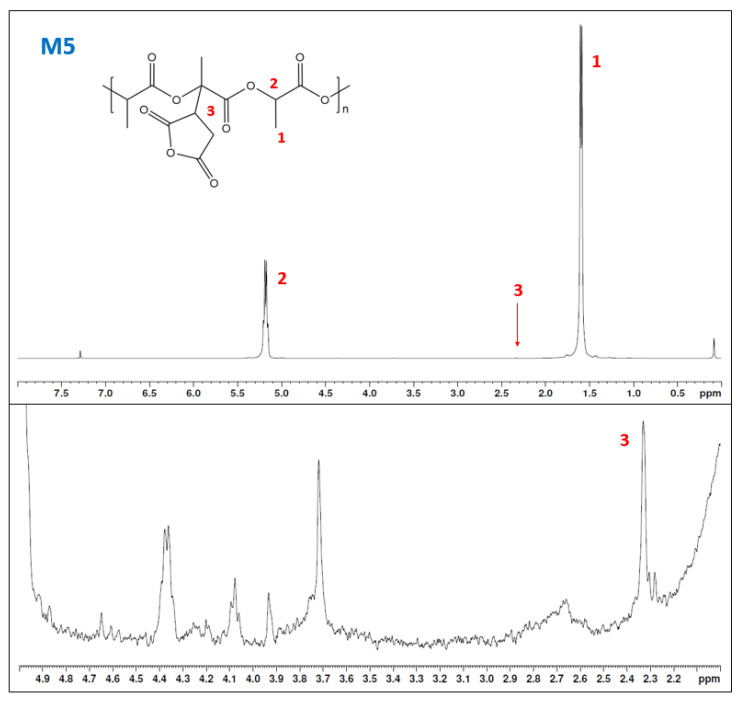
H^1^-NMR spectra of sample M5 (upper) and magnification (down) of the 2–5 ppm displacement in sample M5 (C).

GPC values reported a slight reduction in the molecular weight of the maleated samples and small differences in the PDI value. Additionally, a small degradation could be expected due to the temperature, and in the case of M4, the presence of oxygen in the reaction. Nonetheless, the values of grafting and the lower degradation indicated the benefits of the solvent system [28]. Moreover, the effect of oxygen inhibition and enhanced degradation of PLA is represented in sample M5. Nonetheless, although the GPC and the NA results led us to consider the presence of some grafting in the sample M5, NMR is necessary to confirm the hypothesis. The NMR of sample M5 and the magnified spectra in the range from 2 ppm to 5 are shown in Figure 7.

H^1^-NMR spectra of the sample M5 showed the main peaks of PLA and a disruption in the baseline. When this disruption is magnified (bottom part of Figure 7), some peaks appeared. The peak at 2.33 ppm is associated with the methine hydrogen in the succinic ring. Some authors have related the peak at 3.73 ppm to the -CH_2_- of the succinic ring, but it could be also crosslinking [26,29,30,31]. Peaks in the range of 4.0 to 5 ppm are generally devoted to crosslinked PLA or polymerization of the MA [22,32,33,34]. The integral from the NMR of the -CH- from the succinic ring reported a value of 0.14 and 0.15% when the hydrogens from the methyl peak were used as reference. Nonetheless, although the lower GD was obtained from the AN, the H^1^-NMR results confirm that the reaction worked, and optimization of the parameters needs to be performed. Thus, the effects of the time, DCP, and MA parameters were analysed using the AN and the GD (Table 5). 

Three different parameters were analysed: DCP and MA contents and reaction time. It was found that reducing the MA quantity until 5% *w*/*w* produced a reduction in the GD. Nonetheless, when the MA was reduced to 10%, the GD was slightly reduced in comparison with 20% *w*/*w*. Thus, a 10% MA was considered the optimal quantity in terms of graft efficiency and costs. The DCP quantity was studied by the addition of 1 and 2% *w*/*w*. Lower quantities were discarded due to the poor effect in the extrusion reaction. Again, a small difference was observed between both grafting reactions, and 1% *w*/*w* DCP was used for the coupling agent synthesis. After the observed small effects with the quantities of the reagents, time was considered critical to obtain a good maleation of PLA. The reaction was carried out for 4, 8, 24, and 96 h to analyse its effect. At short times, 4 and 8 h, differences in the graft were not significant. However, the effect of time is appreciated with a longer reaction time, obtaining a maximum GD of 3.5% at 96 h. The formulation M11, which achieved that GD of 3.5%, was considered enough to be used as a coupling agent and was characterized. 

**Figure 8:**
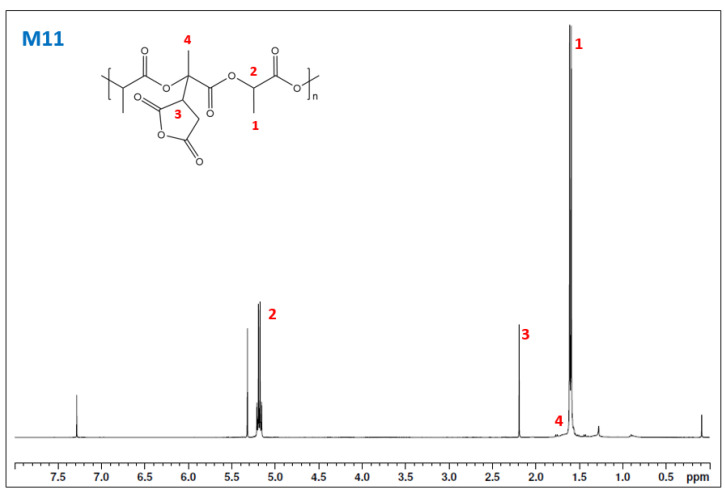
H^1^-NMR spectra of sample M11.

The ^1^H-NMR spectra were used to confirm the grafting in the chemical structure of PLA. The M11 spectra (Figure 8) presents the clear presence of grafting in the polymer structure with peak 3 at 2.3 ppm. An unexpected peak was shown at 5.3 ppm, which some authors related to a rearrangement in the polymer chain [35]. Nonetheless, it could be contamination of dichloromethane [36].

**Figure 9:**
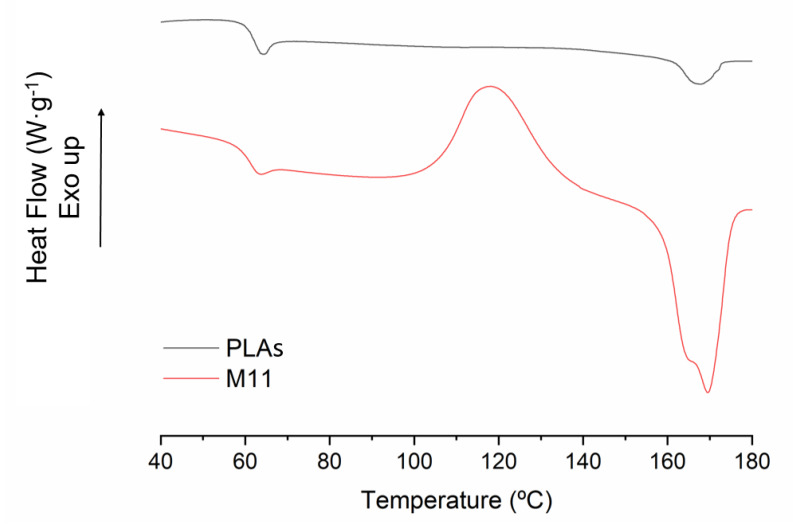
DSC thermograph of the second melting process of PLAs (black) and M11 (red). Exothermal processes are represented in positive.

The thermal behaviour of the sample was analysed using DSC. The thermogram of the second melting of the neat PLA and the M11 sample is shown in Figure 9. PLA shows a glass transition temperature (T_g_) at 61 °C (Table 6) and a reduced melting temperature due to the poor crystallization kinetics of PLA. The grafted sample showed the same T_g_ temperature (61 °C), however, in the coupling agent, the sample showed a clear cold crystallization at 118.3 °C and a broad melting peak with two overlapped peaks. The main peak of melting with the higher intensity was found at 169.4 °C, which is 2 °C shifted to higher temperatures than in the neat PLA (167.5 °C). The other peak temperature is around 165.4 °C. This double melting behaviour observed in sample M11 led us to consider the presence of a grafted part in the polymer. Regarding the crystallization behaviour, it is not clear what the effect of grafting in crystallization is. Some authors have found a reduction in it while others observed a slight improvement [22,37]. Nonetheless, in all the previously published cases, the grafting degree was lower than 1% and obtained by reactive extrusion, while in the analysed sample, the GD was over 3%. The reduction in the Mw during the grafting process could act as a nucleation agent. Moreover, the same effect with lower intensity was also observed for the other samples produced in this work. 

### 3.2. Coupling Agent Performance in Composite Materials

As above mentioned, the objective of the synthesis of the PLA coupling agent is to increase the miscibility of the PLA matrixes and the reinforcement of the composite material. The improvement of the miscibility and the capacity to stablish strong intermolecular interactions between the polymer matrix and the reinforcement phase could assure a correct stress-transfer transmission. Thus, an increment in the tensile strength is expected due to the better stress transfer from the thought phase (the polymer) to the more resistance phase (the natural fibres). The efficiency of the synthesized coupling agent was studied by the production and characterization of composite materials reinforced with 10% of lignocellulosic fibres and using 4% of the coupling agent. Previous studies in the group showed improvements in the tensile strength of composite materials when MAPP or MAPE was up to 4% *w*/*w* polymer [38]. Thus, as the main objective of the test is to analyse the efficiency of the coupling agent, the minimum quantity was used. The results obtained are shown in Table 7. An improvement is observed by the addition of the reinforcing fibres into the composite material. This effect has been previously observed with natural and man-made fibres [39]. The addition of 10% of lignocellulosic fibres represented an improvement of 15%. The reinforcement effect of the natural fibres is improved by the addition of the coupling agent. In that case, the improvement increased up to 24%. It is commonly accepted that the improvement of tensile strength in composite materials is due to good dispersion and strong interfaces. Thus, the improvement in the composite material suggested that the prepared coupling material interacts with the cellulosic fibres, enhancing the miscibility and improving the interface through stronger interactions. 

An increment in the composite stiffness is observed due to the introduction of fibres which are of a stiffer phase than the polymer. Nonetheless, slight differences are observed between both materials. Stiffness is considered to be slightly affected by the strength of the interface and has a higher dependence on fibre dispersion. The results indicate that an adequate dispersion was obtained during the compounding of the material produced. Finally, deformation could be enhanced by the presence of the coupling agent. The addition of fibres generally produces a reduction in the composite deformation, which could be attenuated by a strong interface. However, PLA is a highly rigid and fragile material that generally shows quite poor deformation at room temperature. The effect of the coupling agent could be covered up due to the high fragility of both phases. Nevertheless, the clear improvement in composite tensile strength at nonoptimized quantities demonstrated the efficiency of the coupling agent. 

Micromechanics models are used to evaluate the contributions of the phases to the mechanical properties of the composites. In the case of semi-aligned short-fibre-reinforced composites, the contribution of the reinforcements to any mechanical property is impacted, at a greater or lower level, by the properties of the phases, their percentages, the length and diameter of the reinforcements as well as their dispersion, mean orientation and angle concerning the loads and the compatibility between phases, which will affect the strength of their interface. Modified rules of mixtures (mRoM) for the strength and Young’s modulus of such composites have been successfully used in the literature to evaluate the aforementioned contributions [21,40,41,42,43,44]. Such mRoMs have the following formulation:

(3) $$\sigma^{C}=f_{c}\cdot \sigma^{F}\cdot V^{F}+\sigma^{M*}\cdot V^{M}$$

(4) $$E^{C}=\eta_{e}\cdot E^{F}\cdot V^{F}+E^{M}\cdot V^{M}$$

In Equation (3), an mRoM for the tensile strength, $\sigma^{C}$,$\;\sigma^{F}$, and $\sigma^{M*}$ are the tensile strength of the composite, the intrinsic tensile strength of the reinforcement, and the corresponding stress of the matrix at the strain at break of the composite, respectively. The $\sigma^{M*}$ is recovered from its stress–strain curve. The fibre and matrix volume fractions are represented by $V^{F},$ and $V^{M}$, respectively. If voids are discarded, $V^{M}=1-V^{F}$. Finally, $f_{c}$ is a coupling factor, with values ranging from 0 to 1, that lessens the potential contribution of the reinforcement due to aspects related to its morphology, mean orientation and the strength of the interface.

Equation (4) is an mRoM for Young’s modulus. In the equation, $E^{C}$,$\;E^{F}$ and $E^{M}$ are Young’s moduli of the composite, the reinforcement, and the matrix, respectively. The modulus efficiency factor ($\eta_{e}$) has a similar role as the coupling factor but does not account for the strength of the interface, and as such, strength has been reported to have little impact on Young’s modulus of the composite [43,45].

**Figure 10:**
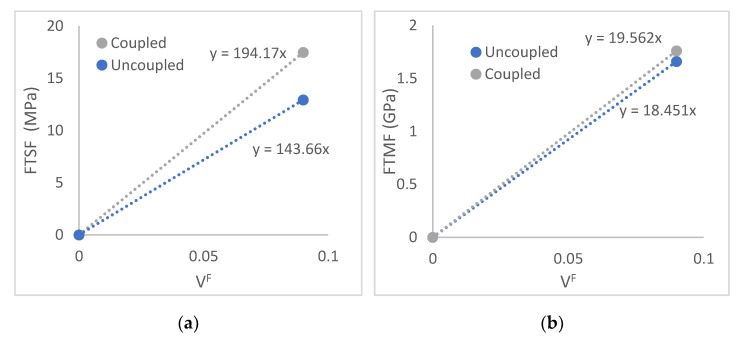
Net contributions of the reinforcements to the tensile strength and Young’s modulus of the composites, (**a**): tensile strength, (**b**): Young’s modulus.

Equations (3) and (4) present two unknowns, $f_{c}\cdot \sigma^{F}$ and $\eta_{e}\cdot E^{F}$. Thus, it is not possible to directly obtain a result for the intrinsic properties of the reinforcements or the efficiency of the contribution of such reinforcements to the property of the composite. Nonetheless, the aforementioned unknowns are the net contributions of the fibres to the strength and Young’s modulus of the composite, and have been previously defined as fibre tensile strength factor (FTST) and fibre tensile modulus factor (FTMF), respectively. These factors evaluate the contribution of the reinforcements against their volume fractions (Figure 10).

Figure 10a shows how the contribution of the fibres with the inclusion of the coupling agent increases noticeably concerning the rest of the composites. FTSF, read as the slope of the regression line, shows how the potential contribution due to the presence of coupling agents will predictably increase for higher fibre volume fractions. These FTSF are similar to uncoupled cotton fibres such as polypropylene reinforcement, with an FTSF of 136.2. Coupled cotton fibres showed a 176 FTSF, lower than the 194 obtained for TMP as PLAc reinforcement [46,47]. Glass fibre as PP reinforcement shows an FTSF of 250. This is due to the higher intrinsic properties of glass fibre. On the other hand, coupled TMP from orange tree pruning returned a FTSF of 98, lower than the found for TMP as PLAc reinforcement. Thus, coupled TMP fibres show a high strengthening potential, only 22% lower than glass fibre as PP reinforcement.

In the case of Young’s modulus, the effect of the coupling agent is not as relevant as in the case of the tensile strength. The coupling agent is responsible for increasing the strength of the interface, and such a factor has a limited effect at the deformation where Young’s moduli are measured. Compared to other reinforcements such as cotton for PP, TMP show higher FTMFs. Coupled and uncoupled cotton fibres showed FTMFs of 13.8 and 12.0 [48]. On the other hand, glass fibres show their stiffening capabilities with a 32.8 FTMF. 

Semialigned, short-fibre-reinforced composites with strong interfaces show coupling factors in a range between 0.18 and 0.20 [5,18]. If these values are used with Equation (1), it is possible to obtain a value for the intrinsic tensile strength of the reinforcement. In any case, the intrinsic tensile strength of the reinforcement will be equal to or higher than the obtained value. The highest value of 1079 MPa can be obtained with a coupling factor of 0.18 and the experimental values of the coupled composite were used in the calculus. If this intrinsic strength is used with the experimental values of the composites reinforced with BKF, a 0.13 coupling factor is obtained. This value shows the existence of interactions between the phases but is indicative of weak interfaces. Thus, the use of a coupling agent had a relevant role in strengthening the interface and also in exploiting the strengthening capabilities of the reinforcement.

In the case of the intrinsic Young’s modulus of the reinforcement, Hirsh’s model has proved efficient to obtain such a value:

(5) $$E^{C}=\beta \cdot (E^{F}\cdot V^{F}+E^{M}\cdot V^{M}+\left(1-\beta \right)\frac{E^{F}\cdot E^{M}}{E^{F}\cdot V^{F}+E^{M}\cdot V^{M}}$$

In this equation, β equalizes the contribution of the fibers as a combination between parallel and series models. This factor takes a 0.4 value for semialigned, short-fiber-reinforced composites [43,49]. The intrinsic Young’s moduli for coupled and uncoupled were 36.2, and 37.5 GPa, respectively. If these values are used with Equation (4), the corresponding modulus efficiency factors are 0.51, and 0.52, respectively. These values are inside the 0.45 to 0.55 expected range [50]. 

## 4. Conclusions

A coupling agent for PLA was synthetized and tested with cellulosic fibres. The synthesis of the coupling agent must be performed in a solvent system and under an inert atmosphere (N_2_). The optimized conditions for the synthesis were: 1% DCP, 10% MA, and 96 h of reaction. The use of a 4% of coupling agent in PLAc + 10% BKF composites produced an increment in the tensile strength of around 24% in comparison with the composite without coupling agent that produced an increment of 15%. The increment in the tensile strength is usually related to the presence of a correct interphase. No effect is observed on the Young’s modulus and deformation, but this could be related to the stiffening effect of the reinforcement fibres and the high fragility of the PLAc matrix at room temperature. The micromechanics demonstrates the effect of the coupling agent, achieving a value of 0.18 for the coupling factor of the coupled composites, in the range of the observed for well-bonded composites, and 0.13 for the uncoupled one. The micromechanics of the modulus did not show huge differences, the values of the intrinsic modulus of the fibre and the efficiency factor being slightly superior for the coupled composite. Besides, the difference is too small to be significant, as observed for the micromechanical properties of the composites. The results indicate the improvement of the PLA grafting process, avoiding the degradation of the polymer during the extrusion reaction and enhancing the yield of the reaction, obtaining higher grafting degrees. The effect of the coupling agent has been demonstrated by the mechanical properties of the composites, and could be an interesting alternative for the mixing of other polar phases such as mineral reinforcements or nanonreinforcements. In addition, the coupling agent dosage must be optimized. 

## Figures and Tables

**Table 1:** Synthesis conditions for MA-grafted PLA in solvent synthesis. DCP is dicumyl peroxide, MA is maleic anhydride.

Sample	DCP (% *w*/*w* PLA)	MA (% *w*/*w* PLA)	Time (h)	Nitrogen Purge
M4	2	20	4	NO
M5	2	20	4	YES
M6	1	20	4	YES
M7	1	5	4	YES
M8	1	10	4	YES
M9	1	20	8	YES
M10	1	10	24	YES
M11	1	10	96	YES

**Table 2:** Acid number (AN) and grafting degree (GD) of reactive extrusion samples.

Sample	AN (mg KOH/g PLA)	GD (%)
PLA	9.1 ± 0.7	-
M1	10.9 ± 0.8	0.16
M2	11.0 ± 1.1	0.17
M3	11.1 ± 1.0	0.18

**Table 3:** Acid number (AN) and grafting degree (GD) results of sample M4 and M5 were synthesized in a solvent system without and with an inert atmosphere, respectively.

Sample	AN (mg KOH/g PLA)	GD (%)
PLA	9.1 ± 0.7	-
M4	14.0 ± 0.5	0.39 ± 0.06
M5	25.1 ± 1.6	1.35 ± 0.14

**Table 4:** GPC results of samples M4 and M5. Mn and Mw refer to the average molecular weight and weight-average molecular weight, respectively. PDI is the polydispersity Index.

Sample	Mn (Da)	Mw (Da)	PDI
PLAs	27,800	102,800	3.7
M4	20,700	85,400	4.1
M5	29,200	100,300	3.4

**Table 5:** Acid number (AN) and grafting degree (GD) of the grafted PLA samples prepared for the optimization of the parameters.

Sample	AN (mg KOH/g PLA)	GD (%)
PLA	9.1 ± 0.7	-
M5	25.1 ± 1.6	1.3 ± 0.1
M6	28.4 ± 1.9	1.6 ± 0.2
M7	18.4 ± 1.9	0.8 ± 0.2
M8	28.4 ± 1.9	1.6 ± 0.2
M9	29.2 ± 1.1	1.7 ± 0.1
M10	35.4 ± 1.0	2.3 ± 0.1
M11	45.3 ± 1.6	3.5 ± 0.1

**Table 6:** Temperature transitions obtained from the thermograph: Glass transition temperature (T_g_), cold crystallization temperature (T_c_) and melting temperature (T_m_) The crystallinity index is indicated as α.

Sample	T_g_ (°C)	T_c_ (°C)	T_m_ (°C)	α (%) *
PLA	61.2	-	167.5	6.0
M11	61.2	118.3	165.4/169.4	49.7

* Calculated using 93.6 J/g as the enthalpy for a 100% PLA crystalline.

**Table 7:** Tensile properties of PLAc and PLAc composites with and without the use of the prepared coupling agent. PLAc refers to commercial PLA used for composites, TMP is the thermomechanical pulp used as reinforcement and M11 is the synthetized coupling agent with 10% MA, 1% DCP, nitrogen purge and 96 h.

Sample	Tensile Strength (MPa)	Young’s Modulus (GPa)	Deformation at Break (%)
PLAc	49.8 ± 1.5	3.3 ± 0.1	3.21 ± 0.17
PLAc + 10%TMP	57.1 ± 0.5	4.7 ± 0.1	2.81 ± 0.04
PLAc + 10%TMP + 4%M11	61.7 ± 0.7	4.8 ± 0.1	2.82 ± 0.08
